# Evaluating the effectiveness of the “Spine Up” online program on depression and quality of life in people with multiple sclerosis: a pilot test for a 16-week intervention

**DOI:** 10.3389/fspor.2026.1705531

**Published:** 2026-02-11

**Authors:** Zsuzsanna Szilárd, Irén Sipeki, Renátó Tóth, Tamás Berki, László Tóth

**Affiliations:** 1 Semmelweis University Pető András Faculty, Budapest, Hungary; 2 Apor Vilmos Catholic College, Budapest, Hungary; 3 School of Doctoral Studies, Hungarian University of Sports Science, Budapest, Hungary; 4 Department of Physical Education Theory and Methodology, Hungarian University of Sports Science, Budapest, Hungary; 5 Department of Psychology and Sport Psychology, Hungarian University of Sport Science, Budapest, Hungary; 6 Teacher Training Institute, Hungarian University of Sports Science, Budapest, Hungary

**Keywords:** depression, exercise therapy, multiple sclerosis, online intervention, psychological well-being, quality of life, rehabilitation

## Abstract

**Introduction:**

Depression and reduced physical activity are common challenges among individuals with multiple sclerosis (MS), substantially impacting quality of life. Online, home-based exercise programs may help overcome mobility and access barriers. This pilot study evaluated the feasibility and perceived effects of a 16-week online stabilization-based exercise intervention—the Spine Up Program (SUP)—on depressive symptoms, physical activity, and subjective functional experience in people with MS.

**Methods:**

Fifty participants (mean age = 46.2 years; 86% female), recruited through the Hungarian MS Foundation, completed pre- and post-intervention assessments using the Beck Depression Inventory (BDI), and other socio-demographic and self-related questions. Sessions were held weekly for 50 min online.

**Results:**

No statistically significant changes were observed in depression or physical activity immediately post-intervention. At three months, depressive symptoms showed a small, non-significant reduction. Physical activity remained stable, suggesting prevention of typical decline commonly observed in MS populations. Participants reported increases in confidence, perceived capability, and motivation to engage in movement. Regression analyses indicated that sport before illness and employment status were meaningful predictors of physical activity, whereas no significant predictors emerged for depression.

**Discussion:**

Although quantitative improvements were modest, participants consistently reported positive subjective changes, supporting the feasibility and acceptability of online spinal stabilization training for people with MS. Maintaining physical activity levels and observing even small improvements in mood may hold clinical relevance in a condition characterized by progressive decline.

## 1 Introduction

Multiple sclerosis (MS) is a chronic neurological condition that affects the central nervous system (CNS), leading to both physical limitations and cognitive challenges. The disease occurs due to inflammatory damage to the myelin sheath, which disrupts the transmission of nerve signals and results in progressive neurological deficits. Common symptoms include sudden changes in sensation, muscle weakness, stiffness, and coordination issues, all of which reflect underlying nerve damage (1).

Motor dysfunctions in MS commonly manifest as difficulties with balance, involuntary muscle contractions, impaired motor planning, and persistent pain (2, 3). These issues often lead to gait disturbances, which can restrict independence and diminish the quality of life (QoL) for those affected (4). Among these symptoms, spasticity is particularly significant because it not only causes physical pain but also severely limits daily activities and negatively impacts emotional well-being and social relationships (5). The effective management of symptoms and slowing the progression of the disease therefore require a comprehensive approach that takes into account physical, emotional, and social factors (6). In addition to these visible symptoms, less apparent issues such as fatigue, mood disorders, blurred vision, cognitive impairment, sensory changes, and sexual dysfunction also greatly affect daily living (7, 8). For MS patients, walking can demand higher cognitive effort, which can further exacerbate feelings of fatigue (1).

Depression is a frequent comorbidity in MS, affecting about 25% of patients at any time and up to 50% over a lifetime. Globally, it impacts roughly 5% of the population, with women disproportionately affected, and is projected by the World Health Organization to become the leading cause of disability by 2030 (9). In Hungary, severe depression affects an estimated 600,000–700,000 people annually (10, 11). MS is strongly linked to reduced quality of life, participation, and functional outcomes (12, 13). Mental health care is often hindered by overlapping somatic and psychological symptoms, contributing to under-recognition and undertreatment (14). The unpredictable course of relapses and remissions can further complicate care. While immunomodulatory drugs and corticosteroids can slow disease progression, their effects on fatigue and depressive symptoms are limited (15, 16). Fatigue and depression therefore persist as major challenges, highlighting the importance of non-pharmacological approaches (17).

Exercise and rehabilitation are leading non-pharmacological strategies across the MS disease course. Therapeutic exercise—including aerobic training, robot-assisted gait training, Pilates, and virtual reality—has been shown to reduce depressive symptoms in some studies and to support symptom management and well-being without increasing relapse risk (18–20). Observational work also confirms a strong association between physical activity and reduced depression in MS, with behavioral activation proposed as a key mediator (21). Meta-analytic evidence suggests a moderate depression-reducing effect of exercise, although heterogeneity across interventions indicates the need to identify feasible programs and optimize dose and delivery (22). Consistent movement may additionally foster psychological resilience and social engagement, helping to alleviate stress, anxiety, and depression commonly experienced in MS (23).

Rehabilitation, which includes physical and occupational therapy, is vital for managing MS symptoms. These therapies can enhance coordination, strength, mobility, and cardiovascular fitness, ultimately improving physical function and overall well-being (24–26). Exercise programs can be beneficial even in advanced stages of disability (27). However, sustained benefits depend on ongoing participation. A meta-analysis found that, instructor-led exercise programs could reduced depressive symptoms, regardless of the type of training involved, but that effects did not consistently persist after programs ended, underscoring the importance of interventions that participants can adopt and maintain over time (15).

Remotely delivered and home-based exercise interventions may help overcome logistical and mobility-related barriers in MS rehabilitation. For example, Sosnoff and colleagues (28) found that a home-based exercise program was feasible and safe, and was associated with reduced physiological fall risk in older adults with multiple sclerosis. However, it remains unclear whether a fully online, supervised stabilization-focused program emphasizing trunk control and motor control can be delivered feasibly at home, whether participants find such training acceptable, and whether there is a preliminary signal of benefit for depressive symptoms and activity-related outcomes that would justify a larger controlled trial.

To address these gaps, we developed the Spine Up Program (SUP), an MS-adapted online stabilization-based intervention designed to support postural muscle endurance, core activation, and motor control through structured, supervised sessions delivered in the home environment. The primary aim of this pilot study was to evaluate feasibility and acceptability of delivering SUP online. Secondary, exploratory aims were to estimate the direction and magnitude of change in depressive symptoms (BDI), physical activity (IPAQ), and other patient-reported outcomes. By pairing implementation outcomes with preliminary clinical signals, this pilot study was designed to inform the design of a subsequent adequately powered trial.

## 2 Material and methods

### 2.1 Participants and study design

This study investigated the effectiveness of the SUP, a 16-week online intervention designed for individuals with MS. Participants engaged in live, weekly, group-based sessions conducted individually in their home environment. Each session lasted 50 min and was held online using zoom platform. Prior to participation, each participant's environment was required to be made safe from an accident prevention perspective. The study was approved by the ethical board of Semmelweis University (Ethical number: SE-RKEB: 30/2022). This study employed an exploratory, quasi-experimental pilot design without a control group, aimed at assessing the feasibility and self-reported outcomes of an online, home-based exercise program for individuals with multiple sclerosis. As a pilot study, no *a priori* power calculation was conducted, and the analyses were intended to generate preliminary insights rather than to evaluate clinical efficacy.

The SUP's effectiveness was tested on a cohort of 50 adults diagnosed with MS, recruited through the Hungarian Foundation for Multiple Sclerosis Patients (MSMBA). Eligibility criteria included a confirmed diagnosis of MS, ability to move independently, and no relapses within the two months prior to enrollment. Individuals were excluded if they had recent relapses, surgeries, cognitive or psychological impairments, or other systemic conditions.

### 2.2 Intervention

The aim of the intervention was to strengthen the core muscles, improve motor control, and increase body awareness. The SUP was based on a core-stabilization training concept that incorporated controlled and precise movement patterns. The program offered a variety of exercises specifically designed to improve the functional status of individuals affected by MS, with a strong focus on conscious activation of core muscles during both movement tasks and daily activities. Exercises were primarily performed in sitting, supine, prone, side-lying, and quadruped positions. Emphasis was placed on trunk stability, correct breathing techniques, and the conscious and accurate execution of movements. The exercises were adaptable to all levels of ability, ensuring safe participation for individuals with minimal, moderate, or advanced functional impairments. The program was designed to avoid placing direct, high-intensity strain on the cardiovascular system, thus making it safe for all participants. Sessions were led by a qualified physical education teacher who, as a person with MS, brought personal experience and empathetic support to the group.

Participants completed assessments at two time points: (1) baseline (pre-intervention), collected immediately before the start of the Spine Up program; (2) post-intervention, collected after the completion of the training period. At baseline and post-intervention, participants completed self-reported measures of physical activity (IPAQ), depressive symptoms (BDI), and items evaluating perceived functional changes related to movement and daily activities.

Participants engaged in one supervised online exercise session per week for 16 weeks. The Spine Up Program was developed by a physical education specialist who is also an individual living with MS, combining professional expertise with firsthand experience of the condition. The intervention aimed to improve trunk muscle strength, movement control, and postural stability through core-focused stabilization and coordination exercises performed primarily in sitting, side-lying, supine, and prone positions, ensuring accessibility for individuals with diverse functional abilities. Exercise selection and scheduling were continually adapted to each participant's current condition and individual capabilities, acknowledging the fluctuating nature of MS.

Each session lasted approximately 50 min and followed a consistent structure that began with a warm-up phase incorporating mobility and preparatory movements, progressed into a main phase involving stabilization, strength, coordination, and balance exercises, and concluded with a cool-down period consisting of stretching, relaxation, calming activities, and eye-exercise practices. Prior to the start of the intervention, participants received a thorough introduction to the Spine Up Program and were taught the fundamental principles, including how to identify and activate trunk muscles, maintain intra-abdominal pressure, and coordinate proper breathing. Throughout the program, the instructor provided continuous verbal and visual guidance to support accurate execution of the exercises. Postural control and task performance were monitored closely during each session, and corrections were delivered promptly and professionally when needed. Exercises were performed slowly and deliberately, typically repeated four to six times, with intensity and complexity evolving dynamically in accordance with each participant's functional progress. No cardiovascular-intensive exercises were included. Each session ended with stretching and relaxation to reduce tension and promote recovery. Participants were encouraged to stop exercising immediately if they experienced fatigue, discomfort, or a decline in energy. Overall, the Spine Up Program offered a structured, adaptive, and supportive exercise environment designed to enhance core stability and movement confidence among individuals with MS participating in an online home-based format.

### 2.3 Measures

To improve interpretability, outcomes were assessed at two time points: baseline (Time 1) and post-intervention (Time 2). Depressive symptoms were measured using the Beck Depression Inventory (BDI), and physical activity was assessed with the International Physical Activity Questionnaire—Short Form (IPAQ-SF). In addition, participants completed a study-specific background questionnaire at baseline collecting socio-demographic information and MS-related clinical characteristics. As part of this questionnaire, participants reported the presence of MS-related symptoms by indicating which symptoms they experienced from a predefined list (multiple responses permitted). Reported symptoms were coded into symptom categories and summarized descriptively as frequencies.

#### 2.3.1 The Beck Depression Inventory

The short form of the Beck Depression Inventory (BDI) was used to assess depressive symptoms. This version comprises 9 items and has previously been adapted and validated in the Hungarian context (29). Participants rated each item based on how they had been feeling over the relevant period, selecting one of four ordered response options scored from 0 to 3, with higher scores indicating greater depressive symptom severity. Item scores were summed to yield a total score for analysis.

#### 2.3.2 International Physical Activity Questionnaire—Short Form

The Hungarian version International Physical Activity Questionnaire—Short Form (IPAQ-SF) was used to assess physical activity in this study. The short form of the scale included 7 items and it assesses the frequency (days per week) and duration (minutes per day) of physical activities over the past seven days across three intensity categories**:** vigorous physical activity (e.g., heavy lifting, aerobics, fast bicycling), moderate physical activity (e.g., carrying light loads, slow bicycling, doubles tennis), walking (including at work, at home, for transportation, or leisure). Additionally, it includes an item assessing sedentary behavior, specifically the amount of time spent sitting on a typical weekday. However, we did not use the questions regarding sedentary behavior. Responses were converted into Metabolic Equivalent of Task (MET) values using standardized scoring protocols (30): walking is assigned 3.3 METs, moderate activity 4.0 METs, and vigorous activity 8.0 METs. Multiplying the reported frequency (days per week), duration (minutes per day), and the corresponding MET value yields an overall MET-minutes/week score. Based on these values, respondents are classified into three categories of physical activity: low (not meeting the minimum recommended levels), moderate (≥600 MET-minutes/week), or high (≥3,000 MET-minutes/week) (IPAQ Research Committee, 2005).The IPAQ-SF has been validated in Hungarian context and it showed a reliable tool to assess physical activity (31).

#### 2.3.3 Subjective feelings

Subjective perceptions were assessed post-intervention to capture participants' perceived changes following the program. Participants completed study-specific items rated on 0–10 numeric rating scales evaluating perceived change in (1) capability to engage in sports activities, (2) attitude toward exercise, (3) perceived movement limitations, (4) perceived health condition, (5) ability to perform daily activities, and (6) whether spinal gymnastics achieved its intended goals. Higher scores indicated a more favorable perceived change (or more positive evaluation) on the respective item. Participants could also optionally provide written comments in an open-ended field; these responses were reviewed descriptively. The questionnaire was developed by the research team and demonstrated excellent test–retest reliability (intraclass correlation coefficient = 0.80). The participants could optionally provide written feedback in an open-ended field as well. These free-text responses were exported and reviewed descriptively; recurrent topics were grouped into categories and summarized in the results with illustrative anonymized quotations.

### 2.4 Statistical analysis

Descriptive statistics (means and standard deviations) were computed for all continuous variables, and frequencies and percentages were reported for categorical variables. To examine changes over time in depressive symptoms (BDI) and physical activity (IPAQ), paired-samples t-tests were performed separately for the total sample and by gender (men vs. women). For the total sample, the paired-samples *t*-tests compared BDI scores at Time 1 and Time 2, as well as IPAQ scores at Time 1 and Time 2. The same analyses were repeated for male and female participants to explore potential gender differences in change patterns. Effect sizes for the t-tests were reported using Cohen's *d*. To identify predictors of depressive symptoms and physical activity, two linear regression models were estimated with BDI and IPAQ scores as the dependent variables. Standardized regression coefficients (*β*), *t*-values, and *p*-values were reported for all predictors. Model fit was assessed using R^2^ values, indicating the proportion of variance explained by the predictors.

## 3 Results

### 3.1 Characteristics of the participants

**Figure 1:**
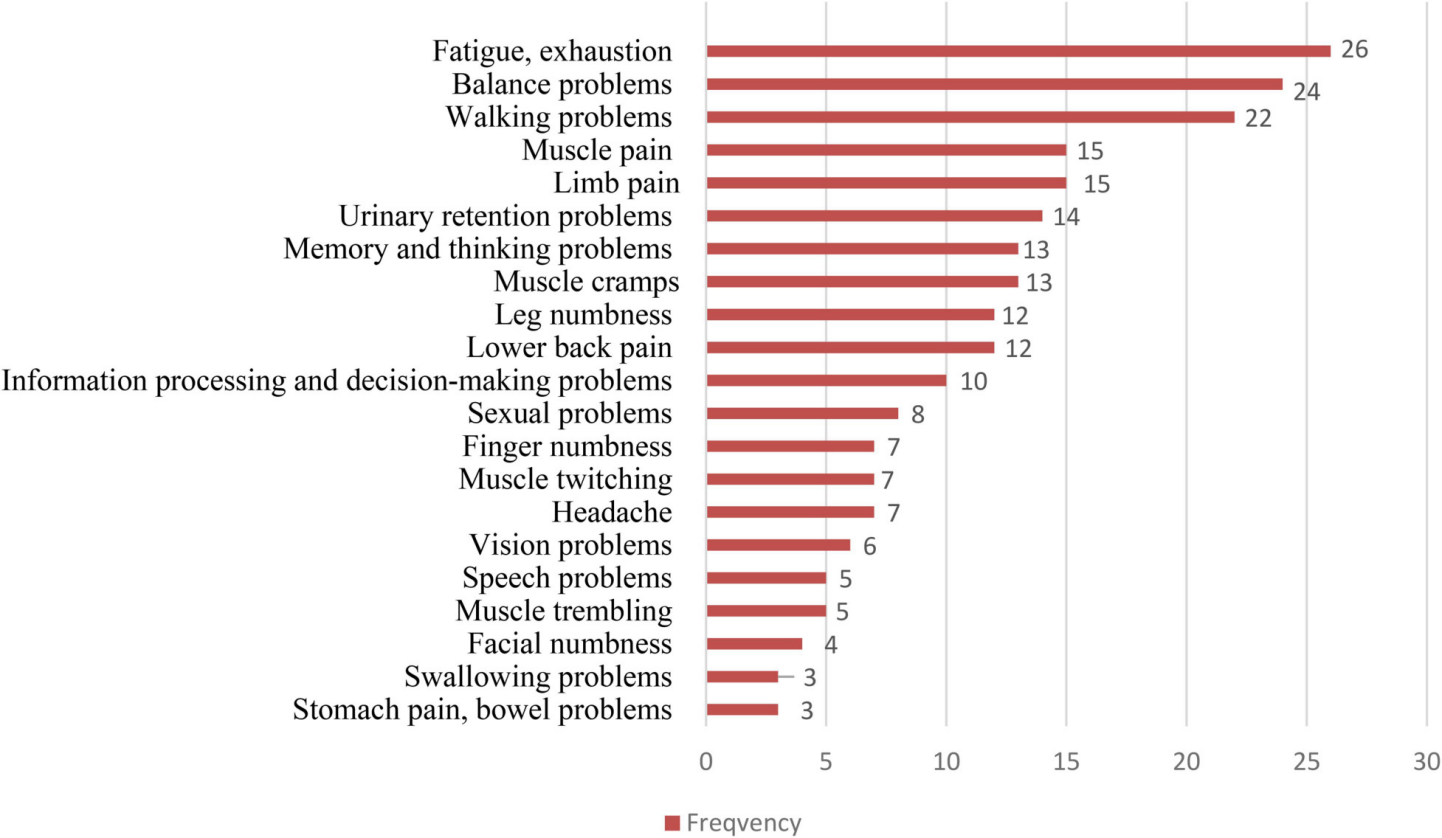
Frequency of symptoms in multiple sclerosis.

Overall, 52 participants participated in this study. Of these, 50 participants completed the full intervention, while 2 participants dropout from the intervention. Reasons for dropout were not recorded. The characteristics of the participants can be found in Table 1. Female (82.0%) were mainly representing the sample (Males = 18.0%). The average participant age was 46.2 years (ranging from 20 to 75), with an average disease duration of 10.4 years (diagnosed between 1987 and 2022). The mean age at diagnosis was 32 years. The majority (64%) were between 40 and 59 years old, with 28% younger than 40. Most individuals had attained tertiary education (74.0%), while 26.0% reported secondary-level education. Before the onset of illness, 92.0% of participants were employed; at the time of data collection, 50.0% remained employed. Pain since illness onset was reported by 76.0% of participants. More than half engaged in sports prior to illness (58.0%), but only 36.0% continued to participate in sports afterward. The study included a diverse group of MS patients, both newly diagnosed and with long-term illness (Figure 1). A majority were receiving medication at the time of the study (64.0%), and 18.0% reported participating in rehabilitation services. Participants identified a total of 21 distinct symptoms related to multiple sclerosis, with fatigue and general exhaustion reported most frequently. These were followed by issues affecting balance and walking ability. Less commonly mentioned symptoms included difficulties with swallowing and gastrointestinal complaints, illustrating the wide spectrum of MS manifestations experienced by the study group.

**Table 1:** The main characteristics of the sample (*n* = 50).

Variable	Category	*N*	%
Gender	Female	41	82%
	Male	9	18%
Education level	Secondary	13	26%
	Tertiary	37	74%
Receiving medication	Yes	32	64%
	No	18	36%
Rehabilitation	Yes	9	18%
	No	41	82%
Worked before illness	Yes	46	92%
	No	4	8%
Currently working	Yes	25	50%
	No	25	50%
Pain since onset	Yes	38	76%
	No	12	24%
Sport before illness	Yes	29	58%
	No	21	42%
Sport after illness	Yes	18	36%
	No	32	64%
Disease status	Stable	34	79%
	Worsening	7	16%
	Improving	2	5%

### 3.2 Gender comparison and the intervention effects

Paired-samples *t*-tests were conducted to examine changes in depressive symptoms (BDI) and physical activity levels (IPAQ) across two time points (Table 2). In the total sample (*N* = 43), depressive symptoms showed a small, nonsignificant decrease from Time 1 (M = 12.47, SD = 6.69) to Time 2 (M = 11.19, SD = 7.45), *t*(42) = 1.62, *p* = .113, Cohen's *d* = 0.25. Physical activity levels remained stable from Time 1 (M = 39.93, SD = 8.92) to Time 2 (M = 39.95, SD = 8.19), *t*(42) = −0.02, *p* = .984, Cohen's *d* = −0.00.

**Table 2:** Descriptive statistics and paired-samples *t*-test results for BDI and IPAQ scores (total sample, men, and women).

Group	Measure	Time Point	Mean	SD	*t*(df)	*p*	Cohen's *d*
Total (*N* = 43)	BDI	Time 1	12.47	6.69	1.62 (42)	.113	0.25
		Time 2	11.19	7.45			
	IPAQ-SF	Time 1	39.93	8.92	−0.02 (42)	.984	−0.00
		Time 2	39.95	8.19			
Women (*N* = 37)	BDI	Time 1	12.47	6.69	1.62 (42)	.113	0.25
		Time 2	11.19	7.45			
	IPAQ-SF	Time 1	39.93	8.92	−0.02 (42)	.984	−0.00
		Time 2	39.95	8.19			
Men (*N* = 6)	BDI	Time 1	11.50	5.47	0.00 (5)	1.000	0.00
		Time 2	11.50	7.20			
	IPAQ-SF	Time 1	38.83	8.52	1.08 (5)	.329	0.44
		Time 2	35.50	6.41			

BDI, Back Depression Inventory; IPAQ, International Physical Activity Questionnaire-Short Form.

A similar pattern was observed among women (*N* = 37). BDI scores decreased slightly from Time 1 (M = 12.47, SD = 6.69) to Time 2 (M = 11.19, SD = 7.45), *t*(42) = 1.62, *p* = .113, Cohen's *d* = 0.25, while IPAQ scores remained effectively unchanged between Time 1 (M = 39.93, SD = 8.92) and Time 2 (M = 39.95, SD = 8.19), *t*(42) = −0.02, *p* = .984, Cohen's *d* = −0.00. In contrast, the men's subgroup (*N* = 6) showed no change in depressive symptoms, with identical means at Time 1 (M = 11.50, SD = 5.47) and Time 2 (M = 11.50, SD = 7.20), *t*(5) = 0.00, *p* = 1.000, Cohen's *d* = 0.00. However, physical activity declined from Time 1 (M = 38.83, SD = 8.52) to Time 2 (M = 35.50, SD = 6.41), although this change did not reach statistical significance, *t*(5) = 1.08, *p* = .329, Cohen's *d* = 0.44.

### 3.3 Subjective feelings after intervention

Results also demonstrated a modest but consistent improvement in attitudes toward physical activity across all groups, that spinal-focused exercises like SUP can enhance motivation, confidence, and willingness to remain active among people with MS (Table 3). Participants rated their experiences between 7 and 10 on a 10-point scale, reflecting generally favorable impressions. Many reported feelings more capable during everyday tasks and more confident in staying active. Those who experienced symptom improvement gave the most positive feedback, noting greater involvement in daily routines and stronger alignment of the program with their health and lifestyle goals. Participants with stable or improving conditions tended to form a more optimistic view of exercise, reporting gains in self-esteem and motivation, especially when they had set personal targets at the beginning of the program. The program appeared particularly effective when combined with medication or rehabilitation therapies. Even participants who had previously reduced their activity found renewed engagement, reporting that they felt more in control of their bodies.

**Table 3:** Post-intervention patient-reported perceived changes and program satisfaction (*n* = 43).

Please rate the extent of positive change you experienced since completing the Spine Up Program for each item (0 = no positive change; 10 = very large positive change).	Mean	SD
Since completing the program, how much more capable do you feel of engaging in sports or exercise activities?	7.2	2.37
Since completing the program, how much has your attitude toward exercise changed in a positive direction?	7.8	2.13
Since completing the program, how much have your movement limitations changed in a positive direction?	7.5	2.53
Since completing the program, how much has your overall health condition changed in a positive direction?	6.9	2.40
Since completing the program, how much has your ability to perform daily activities changed in a positive direction?	6.2	2.83
Overall, to what extent did the spinal stabilization exercises (spinal gymnastics) achieve their intended goals?	7.0	2.40

Higher scores indicate greater perceived positive change.

Only five participants answering the optional post-intervention open-ended comment field, they shared the following: “Daily tasks are easier now.”; “Exercising from home saves energy.”; “I can feel parts of my body again.”; “By the end of the session, the day's pain had eased.”; “My low mood lifted after the session.”; “I'm energized by the group atmosphere.”

### 3.4 BDI and IPAQ predictors

Table 4 presents the results of two linear regression models predicting BDI and IPAQ scores. The regression model predicting BDI demonstrated a small-to-moderate association with the outcome, R = .38, R^2^ = .14, indicating that 14% of the variance in BDI scores was explained by the included predictors. None of the predictors reached statistical significance, although sport before illness showed a trend-level negative association with BDI (*β* = −0.26, *t* = −1.51, *p* = .001). The regression model predicting IPAQ scores showed a stronger association with the outcome, R = .48, R^2^ = .23, explaining 23% of the variance. In this model, several predictors showed meaningful associations. Sport before illness negatively predicted IPAQ scores (*β* = −0.10, *t* = −0.54, *p* = .032), while currently working was positively associated with physical activity (*β* = 0.25, *t* = 1.52, *p* = .001). All remaining predictors demonstrated nonsignificant effects.

**Table 4:** Results of the linear regression of physical activity and depression variables. .

Predictor	BDI			IPAQ-SF		
	*β*	*t*	*p*	*β*	*t*	*p*
Rehabilitated (0/1)	−0.09	−0.56	.581	−0.03	−0.18	.859
Disease status (1–3)	−0.11	−0.65	.521	−0.15	−0.92	.363
Sport since illness (0/1)	−0.09	−0.53	.597	−0.12	−0.76	.454
Sport before illness (0/1)	−0.26	−1.51	.001	−0.10	−0.54	.032
Currently working (0/1)	0.18	1.05	.042	0.25	1.52	.001
Worked before illness (0/1)	−0.00	−0.00	.998	0.05	0.29	.773
Education level (0/1)	−0.11	−0.60	.553	−0.19	−1.07	.293

BDI, Back Depression Inventory; IPAQ, International Physical Activity Questionnaire-Short Form; Codes reflect binary variables where 0 = No and 1 = Yes.; Disease status is coded as 1 = stable, 2 = worsening, 3 = improving; 0 = Secondary education, 1 = Tertiary education.

## 4 Discussion

The primary aim of this pilot study was to evaluate the feasibility and perceived impact of the online, home-based Spine Up Program (SUP) for individuals with multiple sclerosis (MS). Our, results suggest that the program was well-tolerated and perceived positively, with several participants reporting improvements in daily functioning and attitudes toward movement. A small, non-significant reduction in depressive symptoms was observed at follow-up, and physical activity levels remained generally stable across time. While these findings provide preliminary insight into the potential value of an online movement program for people with MS, they should be interpreted cautiously given the study's exploratory design and methodological limitations. This program is designed to improve trunk stability and encourage intentional core engagement during daily tasks.

Physical activity is a recognized non-drug intervention that plays a vital role in the comprehensive management of MS (22). It provides both measurable health benefits and significant psychosocial advantages. Regular participation in physical activity helps manage symptoms, supports cardiovascular fitness, may slow disease progression, and improves the overall quality of life for individuals with MS (20). Effective management goes beyond just exercise; it also requires a balanced lifestyle that includes proper nutrition, smoking cessation, stress management, preventive healthcare, and the management of coexisting health conditions. After the intervention IPAQ scores remained largely stable across the intervention period, and the small decrease observed at follow-up was not statistically significant. This lack of significant decline is noteworthy, as individuals with MS typically show a progressive reduction in physical activity over time due to fatigue, mobility challenges, and symptom fluctuations (32). Therefore, maintaining physical activity levels can itself be considered beneficial. The stability of IPAQ scores in this study suggests that the Spine Up Program may have supported participants in sustaining their existing activity routines rather than experiencing the downward trajectory commonly seen in MS populations. This interpretation is consistent with participants' subjective reports indicating increased confidence and more positive attitudes toward movement (32). However, because the observed changes were small and non-significant, these findings should be interpreted cautiously.

Regarding depressive symptoms, participants showed a small reduction in BDI scores after the intervention; however, this change was not statistically significant and must be interpreted cautiously given the study's uncontrolled, exploratory design. Depression is highly prevalent in MS and strongly associated with reduced quality of life, lower physical activity, and greater functional impairment (33, 34). Exercise interventions have been shown to produce modest improvements in depressive symptoms in MS, with meta-analytic evidence suggesting meaningful but variable effects across different program types and intensities (35). Although the present study did not demonstrate significant changes, the slight downward trend in depressive symptoms is consistent with prior research indicating that regular physical activity and structured movement programs may support psychological well-being in people with MS. Given the absence of a control group and the lack of statistical power, the current findings should be viewed as preliminary. Future studies using larger samples and randomized controlled designs are needed to evaluate whether online, home-based programs like Spine Up can produce reliable improvements in mood outcomes for individuals with MS.

Although only five participants completed the optional post-intervention open-ended comment field, their remarks add useful context to the quantitative findings and point to mechanisms that may support engagement and perceived benefit. Participants described meaningful functional and experiential changes (“Daily tasks are easier now,” “I can feel parts of my body again”), symptom relief (“By the end of the session, the day's pain had eased”), and psychological effects (“My low mood lifted after the session,” “I'm energized by the group atmosphere”). These patient-perceived improvements align with evidence from systematic reviews showing that exercise in MS can yield modest but clinically relevant gains in domains that matter to daily life—particularly balance, walking-related outcomes, fatigue, and quality of life. Notably, one participant emphasized feasibility and energy conservation (“Exercising from home saves energy”), which echoes growing evidence that home-based and tele-exercise formats can be acceptable and may improve fatigue, mood, and broader health-related outcomes in MS. t the same time, these written comments were descriptive and were not collected using a formal qualitative interview protocol; the small number of respondents also limits transferability and increases the likelihood of response bias. Still, the presence of “group atmosphere” in participants' feedback is consistent with qualitative work suggesting that community or group-based exercise can foster social support and positive experiences that help people with MS maintain participation (36).

This study has several limitations. The most significant is the pre-test/post-test design without a control group, which prevents causal conclusions about the effects of the Spine Up Program (SUP). Reductions in depressive symptoms may be attributable to natural symptom fluctuation in MS, regression to the mean, the passage of time, or non-specific factors such as social engagement and increased attention, rather than the intervention itself. Second, the study was exploratory in nature and not powered for effectiveness analyses. No *a priori* sample size calculation was conducted, and the small sample size—particularly in the male subgroup—limits statistical power and the stability of the estimates. Third, the study relied entirely on self-report measures. Although the SUP was designed to target motor control and postural endurance, no objective physiological or performance-based assessments were included, limiting conclusions about the program's physical effects. Additionally, participants were recruited from a single setting and voluntarily enrolled in an online program, which may limit generalizability to the broader MS population. Individuals with higher motivation or digital literacy may be overrepresented. Despite these limitations, the study provides useful preliminary insight into the feasibility and perceived value of an online home-based exercise program for people with MS. Future studies should use randomized controlled designs with sufficient statistical power, incorporate objective measures of motor and postural function, and evaluate long-term adherence and outcomes across more diverse patient populations. For practitioners—neurologists, physiotherapists, and other MS care providers—these findings reinforce that exercise should be an integral part of standard care. Clinicians can help by encouraging patients to stay active, offering resources, and advocating for accessible rehabilitation services.

## 5 Conclusion

Although conventional statistical significance was not achieved at the 16-week follow-up, participants consistently reported subjective improvements, increased confidence, and positive changes in daily functioning. These self-reported perceived gains suggest that structured spinal exercise programs such as the Spine Up Program (SUP) may offer meaningful real-world support for individuals living with MS, even when measurable clinical changes are modest. As a flexible, home-based intervention, the SUP also highlights the potential value of accessible movement programs that can be integrated into daily life. Future research should build on these preliminary findings by optimizing program content, tailoring interventions to individual needs and functional levels, and incorporating objective performance measures alongside self-report outcomes. Rigorous, adequately powered randomized controlled trials are needed to determine the long-term efficacy of such programs, particularly when delivered through hybrid models that combine in-person and remote formats. Together, this work will help clarify how structured movement interventions can best enhance the physical and psychological well-being of people with MS.

## Data Availability

The raw data supporting the conclusions of this article will be made available by the authors, without undue reservation.
